# Analgesic and Antiinflammatory activity of *Amukkarac curanam*

**DOI:** 10.4103/0250-474X.57296

**Published:** 2009

**Authors:** A. Saraswathy, S. Nandini Devi, R. V. Pradeep Chandran

**Affiliations:** Captain Srinivasa Murti Drug Research Institute for Ayurveda (CCRAS), Anna Hospital Campus, Arumbakkam, Chennai-600 106, India

**Keywords:** *Amukkarac curanam*, Siddha formulation, analgesic and antiinflammatory, pentazocine, indomethacin

## Abstract

*Amukkarac curanam* a polyherbal Siddha formulation was examined for its analgesic and antiinflammatory activity at a dose of 500 mg/kg, p.o. The experimental methods used were tail immersion and acetic acid induced writhing method for analgesic and cotton pellet induced granuloma formation for antiinflammatory activity. Pentazocine (10 mg/kg, intraperitoneally) and aspirin (150 mg/kg, orally) clinically used analgesics were used as standard analgesics. Indomethacin (10 mg/kg, orally) was used as standard for antiinflammatory study. *Amukkarac curanam* showed significant analgesic and antiinflammatory activity in the above study.

Conventional or synthetic drugs used in the treatment of diseases are sometimes inadequate and can have serious adverse effects. There is a world wide trend to search for traditional medicines. Siddha medicare is an ancient system of medicine popular amongst Tamil speaking world practiced for over several thousand years. In the present investigation *Amukkarac curanam* (AC), a polyherbal formulation[1] consisting of medicinal plants is taken for study. Its ingredients and formulation composition are tabulated in Table 1. One of the major ingredients is *Withania somnifera* reported to possess antistress[2], antiinflammatory[3] and immunostimulant[4] properties. Other constituents *Syzygium aromaticum*, *cinnamomum wightii*, *Elettaria cardamomum*, *Piper nigrum*, *Piper longum*[5], *Zingiber officinale* are also reported to be medicinally useful. However scientific data on analgesic and antiinflammatory activity of the formulation is not available. Hence in the present investigation analgesic and antiinflammatory potential of the formulation is explored.

**TABLE 1:** INGREDIENTS OF *AMUKKARAC CURANAM*

Botanical name of the drug	Part used	Quantity (g)
*Syzygium aromaticum* Linn.	Flower buds	1 part
*Cinnamomum wightii* Meiss.	Flower buds	2 part
*Elettaria cardamomum* Maton.	Fruits	4 part
*Piper nigrum* Linn.	Fruits	8 part
*Piper longum* Linn.	Fruits	16 part
*Zingiber officinale* Rosc.	Rhizome	32 part
*Withania somnifera* Dunal.	Tuberous roots	64 part

All the ingredients of *ammukkarac curanam* were procured from Chennai local market and authenticated at Pharmacognosy department of Captain Srinivasa Murti Drug Research Institute of Ayurveda (CSMDRIA). Voucher sample of all the ingredients were preserved in the department. *Amukkarac curanam* (AC) was prepared in pharmacy of CSMDRIA as per the method described in Siddha Formulary of India. Formulation was suspended in 0.5% carboxymethylcellulose (CMC) and administered orally. Carrageenan was procured from M/s. Sigma Chemicals, indomethacin from M/s. Sun Pharmaceuticals Ltd., aspirin from M/s. Ordain Pharmaceuticals Ltd. and pentazocine from M/s. Ranbaxy were used for the study.

Swiss albino mice (20-25 g) were used for analgesic study and adult Wistar rats (150-200 g) for antiinflammatory study. The animals were housed in polypropylene cages in ambient temperature, light/dark cycle, fed on standard diet (M/s Pranav Agro Industries Limited, Sangli, India) and provided water *ad libitum*. Ethical clearance from the Institutional Animal Ethics Committee (IAEC) was obtained prior to performing experiments (IAEC/CSMDRIA/02/2003).

Two different sets of mice were randomized with 3 different groups (n=6) for tail immersion and acetic acid induced writhing test respectively. The groups were: group-1 served as vehicle control and received 0.5% CMC solution, group 2 served as positive control and received either pentazocine 10 mg/kg, intraperitoneally (for tail immersion test) or aspirin 150 mg/kg (for acetic acid writhing test), group 3 served as test group and received the drug at dose of 500 mg/kg body weight suspended in 0.5% CMC. The distal 5 cm of the tail was immersed in the water maintained at about 55°[6]. Time taken for withdrawal response was recorded at 0, 5, 15, 30, 60, 90 and 120 min after administering the drug. The cut off time was fixed at 15 s to prevent injury to the tail. Pentazocine was used as positive control.

Acetic acid 0.25 ml of 1% v/v[7] was given to all groups intraperitoneally after 30 min of the drug administration and onset of writhing was noted down and number of writhing (abdominal contraction, trunk twisting response and extension of hind limbs) were noted down for a period of 15 min. Number of writhings produced in the test groups were compared with standard one[8] and the analgesic activity (in terms of % maximum possible effect) was calculated as, dividing the difference between mean writhes of control group and mean writhes of drug treated group by mean writhes of control group and multiplying it by 100.

The Wistar rats were divided into four groups of 6 each. Pellets of surgical cotton weighing 10±1 mg were sterilized in hot air oven at 120° for 3 h. Four pellets each were aseptically implanted subcutaneously into both axillae and groin region, under light ether anesthesia[9]. The first group served as normal control and the second group served as vehicle control and received the vehicle, 0.5% CMC. The 3^rd^ group received AC suspended in 0.5% CMC, at the dose level of 500 mg/kg body weight, orally for 7 days. The 4^th^ group was treated with indomethacin at 10 mg/kg body weight orally. On 8^th^ day the animals were sacrificed, the pellets were dissected out, cleaned from extraneous tissues and dried in hot air oven over night at 70°. The weight of each pellet was recorded. The weight of the pellets taken out from AC administered rats was compared with the weight of pellets taken out from the control group and from the indomethacin administered rats. Blood samples and liver tissue were collected and biochemical studies were carried out.

Serum and 1% liver homogenate (prepared in double distilled water) were used for the estimation of acid phosphatase[10], glutamate pyruvate transaminase (GPT) and glutamate oxaloacetate transaminase (GOT)[10] and total proteins[11]. Pellet weight was noted in both control and treated animals.

Data were statistically evaluated using ANOVA, expressed as mean±SD followed by Post Hoc Dunnett T3 multiple comparisons test using the 10 version of SPSS computer software. Data were considered significant at *P* < 0.05.

In tail immersion method (Table 2), *Amukkarac curanam* increased latency to thermal stimulation when compared to control. AC was found to exhibit very good analgesic activity. Its analgesic activity is significant when compared to pentazocine.

**TABLE 2:** ANALGESIC ACTIVITY OF AC BY TAIL IMMERSION METHOD

Group	Time taken to withdraw the tail from hot water (s)						
	
	0 min	5 min	15 min	30 min	60 min	90 min	120 min
Control	2±0	2±0	2.25±0.5	1.75±0.5	2.0±0.816	2.0±0.82	2.25±0.5
Pentazocine (10 mg/kg)	6±3.16	8.25±2.36*	8.25±2.06*	9.75±0.5*	9.5±1*	9.5±1*	9±1.15*
AC (500 mg/kg)	2.75±0.5	2.75±0.5	3.75±1.5	4±0.81*	4.25±1.25*	5.25±1.3*	8.5±1.9*

Analgesic activity is compared with untreated control. Values are significant when

* *P*<0.05. Values are mean±SD from 6 animals in each group.

In order to distinguish between the central and peripheral analgesic action of AC, acetic acid-induced writhing response in mice was used. This method is simple, reliable and also affords rapid evaluation of peripheral type of analgesic action. AC showed 73.52% inhibition when compared to aspirin which is 98.32%. There is significant decrease in writhing response induced by acetic acid in AC treated group (Table 3), which proves its analgesic property. The abdominal contraction is related to the sensitization of nociceptive receptors to prostaglandins. It is therefore possible that AC exerts its analgesic effect probably by inhibiting synthesis or action of prostaglandins. Thus the analgesic property of the formulation was demonstrated using both the methods.

**TABLE 3:** ANALGESIC ACTIVITY OF AC BY WRITHING METHOD

Group	Mean of Writhings observed for 15 min	% MPE
Control	79.33 ± 0.57	-
Aspirin (150 mg/kg)	1.3 ± 0.57*	98.32
AC (500 mg/kg)	21 ± 4.58*	73.52

Analgesic activity is compared with untreated control. Values are significant when

* *P*<0.05. Values are mean±SD from 6 animals in each group.

In the antiinflammatory study of AC using cotton pellet-induced granuloma formation, it reduced the activity of GPT, GOT and acid phosphatase activity in serum (Table 4). In liver GPT and GOT activity was reduced by AC (Table 5). AC reduced the activity of acid phosphatase. Protein in both liver and serum were reduced by AC.

**TABLE 4:** SERUM BIOCHEMICAL PARAMETERS OF AC

Group	Protein g/dl	GPT units/dl	GOT units/dl	Acid Phosphatase liberated/dl in 60 min
Control	10.0±0.23	4.80±0.60	10.83±0.83	5.34 ± 0.72
Inflammatory control	11.9±0.96	5.96±0.811*	19.80±1.73*	7.67±0.39*
Indomethacin (10 mg/kg)	10.3±0.02	4.40±0.707*	12.43±1.03*	5.96±0.11*
AC (500 mg/kg)	9.72±0.96*	4.47±0.58*	11.34±1.34*	6.06±0.12*

Inflammatory control is compared with control group and standard and AC groups were compared with inflammatory control group. Values are significant when

* *P*<0.05. Values are mean±SD from 6 animals in each group.

**TABLE 5:** LIVER BIOCHEMICAL PARAMETERS AND PELLET WEIGHT IN RATS TREATED WITH AC

Groups Parameters	Control	Inflammatory Control	Standard Drug (Indomethacin)	Drug treated (AC)
Protein mg/g	86.3±9.6	107.3±11.4*	67.0±4.3	85.7±5.67*
GPT (units/mg/protein)	89.39±0.01	101.35±11.1*	77.27±2.95*	60.67±0.25**
GOT (units/mg/protein)	9.83±0.025	31.91±0.061*	26.51±2.5*	24.38±2.5*
Acid Phosphatase (mg phenol/mg)	0.0375±0.00077	0.0675±0.00091*	0.045±0.0028*	0.0385±0.00071*
Pellet weight (mg)	-	39.56± 4.3	23.62±3.0	32.2±4.0
% reduction in pellet weight	-	-	40.29	18.60

Inflammatory control is compared with normal control and standard and AC is compared with inflammatory control. Values are significant when

* *P*<0.05. Values are mean ± SD from 6 animals in each group.

The inhibition of GOT and GPT activity by this preparation may influence the formation of polypeptides like bradykinin and other kinin-like substances which are released during the inflammatory process. Acid phosphatase is frequently employed as a marker enzyme to assess the lysosomal change during inflammation. By stabilizing the lysosomal membrane, the antiinflammatory drug interferes in the synthesis of lysosomal enzymes which participate in the process of inflammation. Decrease in the levels of protein in serum is indicative of low activity of GPT and GOT by the drug,

AC showed good antiinflammatory activity with 28% reduction in granuloma formation. This method described by Meier *et al.*[12] have showed that foreign body granuloma were provoked in rats by subcutaneous implantation of pellets of compressed cotton. This method has been useful for evaluation of steroidal and non-steroidal antiinflammatory drugs. Cotton pellet-induced granuloma is closely related to the formation of antibodies. AC is found to reduce the antibody formation and thus proves its antiinflammatory property. Based on these results it can be concluded that AC exerted potential analgesic activity, which could probably mediated through both central and peripheral mechanisms. It also has significant antiinflammatory property.
